# Hepatic Myeloid Sarcoma Presenting With Recurrent Ascites: A Case Report

**DOI:** 10.7759/cureus.105880

**Published:** 2026-03-26

**Authors:** Mossaab El Mousadik, Monsif FADI, Zoubida Saad, Najat Lamalmi, Nouama Bouanani

**Affiliations:** 1 Clinical Hematology and Cellular Therapy, Mohammed VI University Hospital Center, Ibn Zohr University, Agadir, MAR; 2 Hematology, Cheikh Khalifa International University Hospital, Mohammed VI University of Health Sciences (UM6SS), Casablanca, MAR; 3 Oncopathology, Cancer Biology, and Environment Laboratory, Mohammed VI Faculty of Medicine, Center for Doctoral Studies (CEDoc), Mohammed VI University of Health Sciences (UM6SS), Casablanca, MAR; 4 Hematology, Faculty of Medicine, Cheikh Khalifa International University Hospital, Mohammed VI University of Health Sciences (UM6SS), Casablanca, MAR; 5 Pathology, Ibn Sina University Hospital Center, Rabat, MAR; 6 Hematology, Faculty of Medicine, Mohammed VI University of Health Sciences (UM6SS), Casablanca, MAR

**Keywords:** acute myeloid leukemia, hepatic malignancy, myeloid neoplasm, myeloid sarcoma, refractory ascites

## Abstract

Myeloid sarcoma (MS) is an extramedullary manifestation of acute myeloid leukemia (AML) that may occur de novo, precede, or accompany AML. Hepatic involvement is rare and poses a diagnostic challenge.

We report a 65-year-old man presenting with progressive abdominal distension, massive ascites, and constitutional symptoms. Initial laboratory tests revealed bicytopenia, leukocytosis, circulating immature granulocytes, and a cholestatic pattern on liver function tests. Imaging showed homogeneous hepatomegaly with large-volume ascites. Liver biopsy demonstrated diffuse infiltration by immature myeloid cells, with immunohistochemical positivity for myeloperoxidase (MPO) and CD117. Bone marrow (BM) cytogenetic analysis revealed an isochromosome 17q, supporting the diagnosis of hepatic MS, associated with an underlying clonal myeloid neoplasm. The patient received azacitidine and venetoclax, with no response, followed by cytarabine and daunorubicin, with transient clinical improvement; however, progression to AML occurred within a few weeks.

This case highlights the diagnostic challenge of hepatic MS presenting as refractory ascites, and supports consideration of early liver biopsy, with sampling from multiple sites, in patients with unexplained ascites and underlying hematologic abnormalities.

## Introduction

Myeloid sarcoma (MS) is an extramedullary manifestation of acute myeloid leukemia (AML) that may occur de novo, precede, or accompany AML, and may also indicate disease progression in myelodysplastic and myeloproliferative neoplasms. MS is suggested to be related to the migration of leukemic blasts to extramedullary tissues, mediated by increased expression of adhesion molecules and chemokines, which promote tissue homing and proliferation [1].

Isolated MS is uncommon, accounting for less than 1% of cases [2], whereas it is associated with AML in approximately 2.5%-9.1% of cases [3-5]. MS can also be classified into two groups, with distinct prognostic implications based on the anatomical site involved: hematopoietic MS (hMS), involving lymph nodes, spleen, and liver, and extramedullary MS (eMS), most commonly affecting the skin, soft tissues, and gastrointestinal tract [6].

The prognosis of isolated MS remains controversial and has been reported to be better than that of synchronous AML in some studies [7]. More specifically, eMS has been associated with improved overall survival compared with hMS [8]. Primary MS is recommended to be treated with the same intensive chemotherapy regimens used for AML, while radiotherapy (RT) or surgery may be considered for symptom control, with limited supporting evidence [9].

We report a case of hepatic MS, initially misdiagnosed as an infectious disease, highlighting the complexity of this rare entity and emphasizing the importance of early biopsy, with sampling from multiple sites, in challenging clinical cases.

## Case presentation

A 65-year-old man, with a history of arterial hypertension and multiple prior inguinal hernia repairs, was admitted for progressive abdominal distension and diffuse abdominal pain of two months’ duration, associated with night sweats and unintentional weight loss. Examination revealed severe anemia, generalized edema, and large-volume ascites.

Initial laboratory tests showed severe cytopenias: hemoglobin 6.2 g/dL, leucocytosis 38,420/mm³ with absolute neutrophilia (18,440/mm³), and thrombocytopenia 56,000/mm³. Peripheral smear demonstrated 41% circulating immature granulocytes. Liver biochemical tests showed a cholestatic pattern, with elevated liver enzymes, while total bilirubin remained within the normal range (Table 1).

**Table 1:** Initial laboratory findings. The reference ranges provided in this table are based on commonly accepted adult clinical laboratory standards and may vary depending on institutional and regional laboratory practices.

Parameter	Result	Reference Range
Hemoglobin (Hb)	6.2 g/dL	13.0-17.0 g/dL (men)
Platelets	56,000/mm³	150,000-400,000/mm³
White blood cells (WBC)	38,420/mm³	4,000-10,000/mm³
Neutrophils (absolute)	18,440/mm³	1,500-7,500/mm³
Circulating immature granulocytes (myelemia)	41%	0% (normally absent in peripheral blood)
Total bilirubin	8 mg/L	3-12 mg/L
Aspartate aminotransferase (AST)	60 UI/L	10-40 UI/L
Alanine aminotransferase (ALT)	69 UI/L	7-56 UI/L
Gamma-glutamyl transferase (GGT)	140 UI/L	9-48 UI/L
Alkaline phosphatase (ALP)	237 UI/L	44-147 UI/L

**Figure 1:**
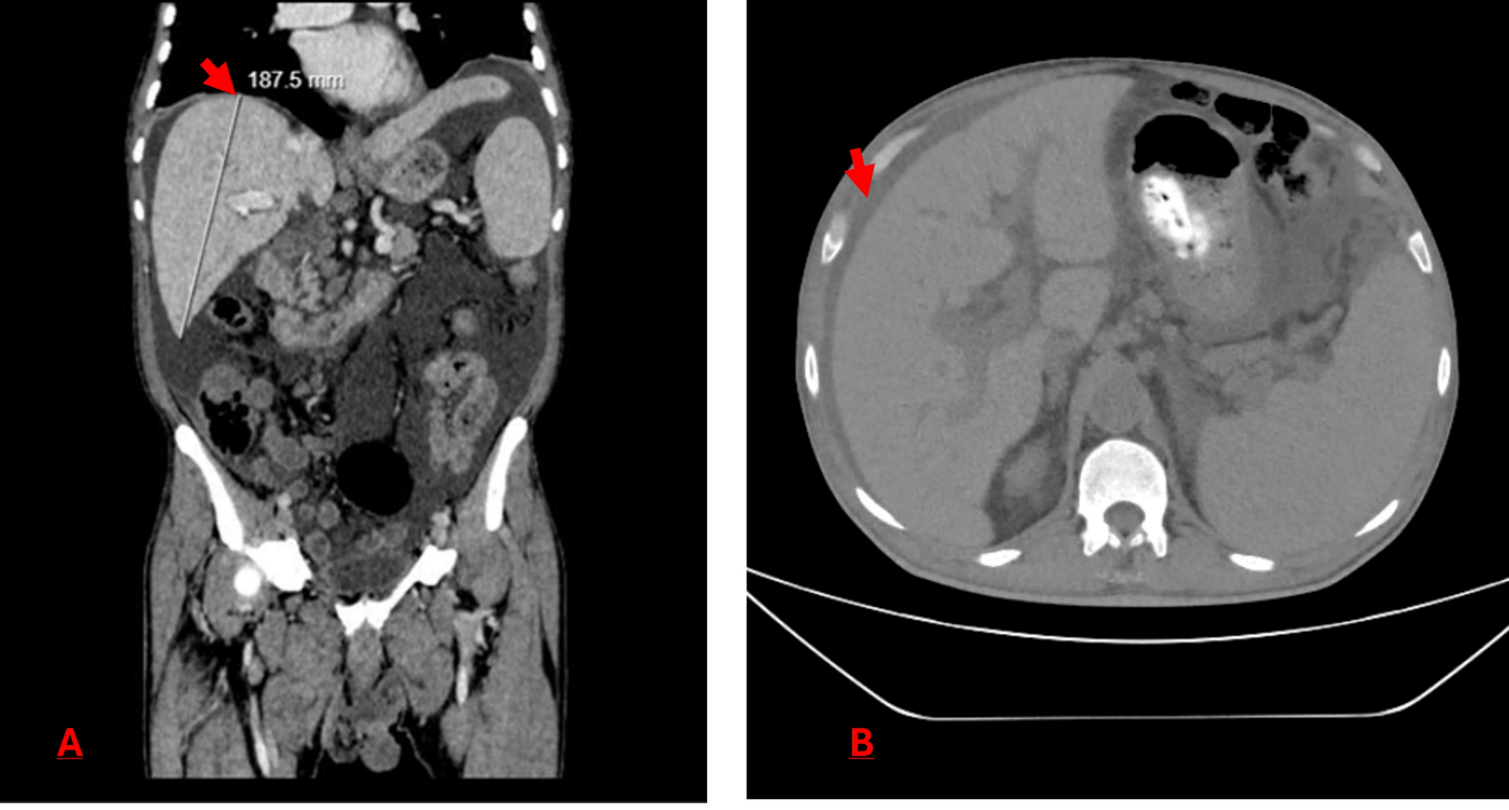
Contrast-enhanced computed tomography of the abdomen. (A) Coronal reconstruction showing marked hepatomegaly (craniocaudal length: 18.7 cm; arrow). (B) Axial image demonstrating perihepatic ascites (arrow).

Bone marrow (BM) aspiration and biopsy revealed hypercellularity with marked granulocytic hyperplasia (81%) and 2% blasts. Immunohistochemistry excluded lymphoid malignancy and AML. Cytogenetics showed isochromosome 17q (46, XY, i(17)(q10)), while BCR-ABL1, JAK2, CALR, and MPL mutations were negative. Overall, these findings were consistent with an atypical clonal myeloid neoplasm. Computed tomography (CT) demonstrated homogeneous hepatomegaly without focal hepatic lesions and large-volume ascites (Figure 1).

Diagnostic paracentesis showed exudative ascites with elevated protein and adenosine deaminase (ADA) levels. Cytology was negative for malignancy, and tuberculosis PCR was negative; however, given the endemic context and elevated ADA levels, empirical antituberculosis therapy was initiated. The patient developed persistent cytopenias, refractory ascites requiring repeated paracenteses, and clinical deterioration, leading to treatment discontinuation after two months.

**Figure 2:**
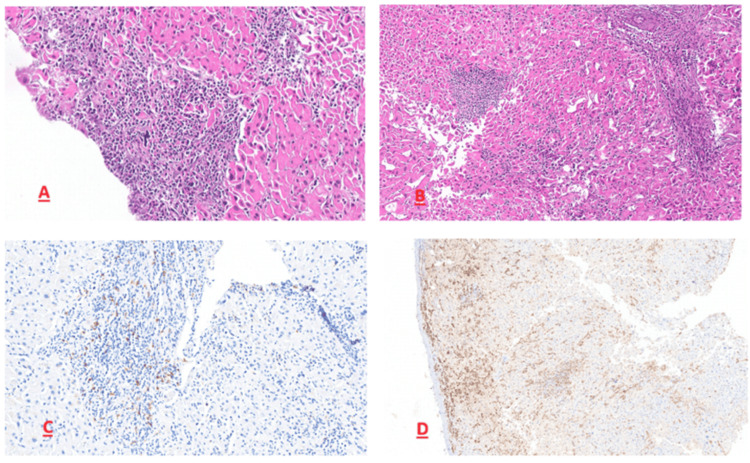
Histopathological and immunohistochemical findings from liver biopsy. (A) Hematoxylin and eosin stain (×10) showing diffuse infiltration of the hepatic parenchyma by immature monomorphic cells with architectural effacement. (B) Hematoxylin and eosin stain (×20) demonstrating tumor cells with a high nuclear-to-cytoplasmic ratio and finely dispersed chromatin. (C) Immunohistochemistry for myeloperoxidase (MPO) showing strong diffuse cytoplasmic positivity in tumor cells. (D) Immunohistochemistry for CD117 (c-Kit) showing focal positivity in tumor cells.

Due to persistent diagnostic uncertainty, exploratory laparoscopy was performed and revealed multiple hepatic nodules not identified on prior imaging. Liver biopsy revealed diffuse infiltration of the hepatic parenchyma by immature monomorphic cells with architectural effacement. Tumor cells exhibited a high nuclear-to-cytoplasmic ratio, finely dispersed chromatin, and a faint nucleolus. Immunohistochemistry demonstrated strong cytoplasmic myeloperoxidase (MPO) expression, associated with focal CD117 positivity, establishing the diagnosis of hepatic MS associated with an underlying atypical clonal myeloid neoplasm after three months of investigations (Figure 2).

Considering the patient’s frail condition, he received one cycle of azacitidine (75 mg/m²/day, days 1-7) combined with venetoclax (400 mg daily, days 1-28) but showed clinical worsening, with transfusion dependence and refractory ascites. Second-line induction chemotherapy with daunorubicin (60 mg/m²/day, days 1-2) and cytarabine (100 mg/m² every 12 hours, days 1-5) achieved transient disease control, with resolution of ascites and improved performance status.

One month later, he was readmitted with recurrent ascites and severe anemia. BM biopsy showed increased blasts, confirming progression to overt AML three months after the diagnosis of MS. Given the poor prognosis, care was transitioned to a palliative approach.

## Discussion

Hepatic MS is extremely rare; it has only been reported in limited case series and is associated with diagnostic difficulty and adverse prognosis [10].

We report an unusual presentation of liver MS. Our patient presented with massive ascites and homogeneous hepatomegaly without hepatic nodules or biliary tract involvement, whereas most of the reported cases described signs of biliary obstruction, right hypochondrium pain, jaundice, and other symptoms mimicking cholecystitis or pancreatitis [10]. Ascites may be explained by diffuse sinusoidal and portal infiltration by leukemic cells, which may disrupt intrahepatic vascular flow and lead to portal hypertension, resulting in ascites [11].

Radiological findings were neither specific nor sensitive in our case, as no focal masses were found, while liver MS is typically described on CT as a less well-circumscribed, hypo-enhancing mass [12]. Therefore, fluorodeoxyglucose positron emission tomography/computed tomography (FDG PET/CT) may be helpful for early detection, radiation therapy planning, and monitoring treatment response [13].

BM involvement can precede MS, be concomitant, or occur within the next 5 to 12 months [9,14]. Histopathological evaluation with immunohistochemistry remains the cornerstone for the diagnosis of MS. In our patient, leukemic cells were positive for MPO and CD117, consistent with pooled data from recent meta-analytic studies demonstrating high expression rates of MPO (~72%) and moderate expression of CD117 (~59%) in MS. Other frequently expressed markers include CD33 (~79%), CD45 (~76%), CD43 (~70%), CD68 (~59%), CD34 (~44%), and lysozyme (55%), reflecting the heterogeneous immunophenotypic profile of this entity [1]. High misdiagnosis rates have been reported in isolated MS when immunohistochemical panels are incomplete, particularly with non-Hodgkin lymphoma [9].

According to WHO 2022, MS, whether isolated or associated with BM involvement, is classified within AML biology [14] and should be treated with AML-type systemic therapy [15]. RT can be used for patients requiring debulking, rapid symptom relief, or in cases of vital structure compression. Some authors have reported using RT as consolidation therapy in isolated MS, without evidence of superiority over aggressive chemotherapy alone [9]. In our case, the lack of response to azacitidine and venetoclax, as well as the transient clinical response to cytarabine-daunorubicin intensive chemotherapy, can be explained by the adverse effect of isolated isochromosome 17q (46,XY,i(17)(q10)) detected in the patient’s initial BM cytogenetic analyses.

Isolated i(17q) is a rare cytogenetic abnormality, occurring in less than 1% of myeloid neoplasms, and it is associated with aggressive disease and poor prognosis. Molecular studies have shown frequent mutations in SETBP1, ASXL1, SRSF2, and RAS pathway genes. The median overall survival after detection of i(17q) is approximately 9.4 months, reflecting its strong association with rapid leukemic progression [16]. In our patient, the presence of i(17q) likely contributed to treatment resistance and an aggressive clinical course.

## Conclusions

Hepatic MS is a rare and diagnostically challenging condition, particularly in the absence of overt AML. It may present with refractory ascites and hepatomegaly, without identifiable focal lesions on imaging. In patients with unexplained ascites and cytopenias, clinicians should consider an underlying infiltrative hematologic malignancy and proceed with a timely liver biopsy. Early biopsy, including sampling from multiple sites, may improve diagnostic accuracy and facilitate a prompt diagnosis. Given the risk of rapid progression to AML, delays in diagnosis can complicate treatment decisions and negatively impact outcomes.
